# Augmented reality in medical education: a systematic review

**DOI:** 10.36834/cmej.61705

**Published:** 2020-03-16

**Authors:** Kevin S. Tang, Derrick L. Cheng, Eric Mi, Paul B. Greenberg

**Affiliations:** 1 The Program in Liberal Medical Education of Brown University, Rhode Island, USA; 2 The Warren Alpert Medical School of Brown University, Rhode Island, USA; 3 Lifespan Clinical Research Center, Rhode Island, USA; 4 Division of Ophthalmology, Warren Alpert Medical School, Rhode Island, USA; 5 Section of Ophthalmology, Providence VA Medical Center, Rhode Island, USA

## Abstract

**Introduction:**

The field of augmented reality (AR) is rapidly growing with many new potential applications in medical education. This systematic review investigated the current state of augmented reality applications (ARAs) and developed an analytical model to guide future research in assessing ARAs as teaching tools in medical education.

**Methods:**

A literature search was conducted using PubMed, Embase, Web of Science, Cochrane Library, and Google Scholar. This review followed PRISMA guidelines and included publications from January 1, 2000 to June 18, 2018. Inclusion criteria were experimental studies evaluating ARAs implemented in healthcare education published in English. Our review evaluated study quality and determined whether studies assessed ARA validity using criteria established by the GRADE Working Group and Gallagher et al., respectively. These findings were used to formulate an analytical model to assess the readiness of ARAs for implementation in medical education.

**Results:**

We identified 100,807 articles in the initial literature search; 36 met inclusion criteria for final review and were categorized into three categories: Surgery (23), Anatomy (9), and Other (4). The overall quality of the studies was poor and no ARA was tested for all five stages of validity. Our analytical model evaluates the importance of research quality, application content, outcomes, and feasibility of an ARA to gauge its readiness for implementation.

**Conclusion:**

While AR technology is growing at a rapid rate, the current quality and breadth of AR research in medical training is insufficient to recommend the adoption into educational curricula. We hope our analytical model will help standardize AR assessment methods and define the role of AR technology in medical education.

## Abstract (fr)

**Contexte:**

Le domaine de la réalité augmentée (RA) est en pleine émergence et dispose de plusieurs nouvelles applications potentielles en éducation médicale. Cette revue systématique a évalué l’état actuel des applications de réalité augmentée (ARA) afin d’et élaboré un modèle analytique pour orienter les futures recherches sur l’évaluation des ARA comme outils pédagogiques en éducationmédicale.

**Méthodes:**

Une recherche documentaire a été menée à l’aide de PubMed, Web of Science, Cochrane Library et Google Scholar. Cette revue a suivi les directives de la méthode PRISMA et contenait les publications du 1er janvier 2000 au 18 juin 2018. Les études étaient retenues si elles avaient un devis expérimental et qu’elles avaient été publiées en anglais et qu’elles évaluaient des ARA mises en place dans l’enseignement des soins de santé. Notre revuea évalué la qualité des études et déterminé si les études ont pu évaluer la validité des ARA en utilisant les critères établis par le GRADE Working Group et Gallagher et coll., respectivement. À partir de ces conclusions, nous avons formulé un modèle analytique afin d’évaluer si les ARA peuvent être mises en place dans la formation médicale.

**Résultats:**

Nous avons trouvé 100 807 articles lors de la recherche documentaire initiale; 36 ont satisfait aux critères d’inclusion pour l’examen final et ont été classés dans trois catégories : chirurgie (23), anatomie (9) et autre (4). La qualité globaledes études était de mauvaise et aucune ARA n’a été testée pour toutes les cinq étapes de validité. Notre modèle analytique évalue l’importance de la qualité des recherches, du contenu des applications, des résultats, et de la faisabilité d’une ARA pour déterminer si elle est prête à être mise en place.

**Conclusion:**

Bien que la technologie de la RA progresse rapidement, la qualité et l’étendue actuelles de la recherche sur la RA en éducationmédicale sont insuffisantes pour recommander son adoption dans le cursus de formation. Nous espérons que notre modèle analytique aidera à uniformiserles méthodes d’évaluations de la RA et à déterminer le rôle de la technologie liée à la RA en éducation médicale.

## Introduction

Over the past decade, augmented and virtual reality technology have demonstrated the potential to transform a variety of fields. Virtual reality (VR) technology creates entirely artificial environments through headsets that isolate users from their surroundings. In comparison, augmented reality (AR) overlays digital interfaces upon physical surroundings, producing an environment that is both real and digital.^1^^,^^2^ This combination of physical and virtual information allows AR to further enhance the well-established methods of procedural simulation.^3^

While the technology and concept of augmented reality have existed for several decades,^4^^,^^5^ recent advances in visual technology and the development of new augmented reality applications (ARAs) have drawn consumer and professional attention.^6^ These applications are software and/or hardware developed explicitly with AR functionality in mind, and have already been applied in many educational settings including environmental sciences, chemistry, humanities, and the arts.^7^^,^^8^ Recent studies have shown that there is a growing number of ARAs in medicine and that AR may foreshadow a new paradigm in medical education.^8^^,^^9^ To date, ARAs have been adapted to every stage of medical training as anatomical teaching tools,^10^ classroom study aids,^11^ image training simulators,^12^ and clinical skills interaction simulators.^13^

This study comprehensively described the use of different ARAs in medical education. Prior systematic reviews have not assessed the quality of recent AR research in medical education and have focused primarily on the integration of surgical ARAs in medical training^9^ or applications in general education.^8^^,^^14^ The purposes of this study wereto conduct a systematic review of the role of AR in medical education, including evaluating the quality of studies and the prevalence of formal validity assessments,^15^^,^^16^ and to develop an analytical model to assess the feasibility of ARA implementation into medical educational curricula.

## Methods

### Systematic review

We conducted a systematic literature search using PubMed, Cochrane Library, Embase, Web of Science, and Google Scholar from January 1, 2000 through June 18, 2018. The Boolean search terms used were “augmented reality” AND (medical education OR medical student OR anatomy education OR surgical education OR surgical training). A university librarian assisted with keyword and database selection to ensure broad coverage that would encompass all existing relevant literature. Search results were recorded per the Preferred Reporting Items for Systematic Reviews and Meta-Analyses (PRISMA) guidelines.^17^

Included articles a) described ARAs in the context of medicine and medical education, b) carried out experimental studies evaluating specific ARAs, c) were obtained from peer-reviewed journals after the year 2000, and d) were written in English. Excluded articles a) discussed VR or similar technologies but not AR, b) were focused on the technological basis for AR or c) discussed AR outside of medicine. Two independent reviewers (D.C., K.T.) conducted the literature search and gathered data, and a third reviewer (E.M.) resolved any conflicts.

Reviewed articles were divided into three categories. “Surgical” applications were designed to train medical novices in procedural tasks such as basic laparoscopic skills, suturing, ventriculostomy, and echocardiography. “Anatomy” applications were designed to assist students with learning human anatomy. “Other” applications were developed for general healthcare education, including clinical skills, forensic medicine, dermatology, and pathology.

### Quality and validity assessment

Studies were assessed for quality using criteria based on the Grades of Recommendation, Assessment, Development, and Evaluation (GRADE) Working Group scoring protocol. Quality analysis was based on metrics including inconsistency in outcomes between different studies, directness of evidence, possibility of bias, confounders, strength of association, dose response, and data quantity.^15^

**Figure 1:**
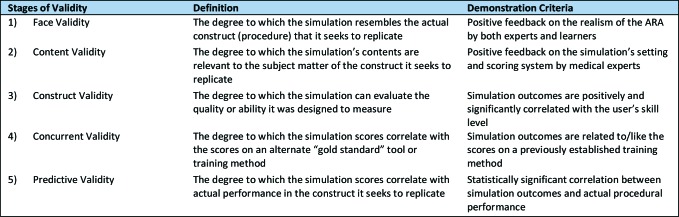
Validity framework overview^9^^,^^16^^,^^18^

In addition to the GRADE quality assessment, we determined whether the included articles assessed ARAs for validity.^16^^,^^18^^,^^19^ This evaluation was informed by Gallagher et al.’s five forms of validity: face, content, construct, concurrent, and predictive validity (Figure 1).^9^^,^^16^ These criteria were initially adopted to evaluate testing instruments in surgical training^16^; more recently, they have been used to validate surgical simulators and their readiness for implementation in surgical curricula.^9^^,^^18^^,^^19^While other validity frameworks have been developed in recent years,^20^^,^^21^ none have been as widely used in evaluating simulation technology in medical education.^9^^,^^16^In order to validate ARAs at any of the five stages, studies were required to either conduct formal validity assessments or demonstrate outcomes that directly aligned with the validity requirements delineated in Figure 1. No specific quantitative variables were analyzed in this review.

### Analytical model

Based on the results of our systematic review, we developed an analytical model to guide future research in assessing the readiness of ARAs for implementation into current medical educational curricula. This model utilized elements from Cook et al.’s approach to evaluating the implementation of technology-enhanced learning (TEL) in medical education as well as the quality criteria described by the GRADE Working Group.^15^^,^^16^^,^^22^

## Results

### Systematic review

We identified 100,807 papers in the initial search. Title screening and removal of duplicates left 439 papers that were evaluated based on abstract. Second-level exclusion removed 347 papers, leaving 93 full-text papers that were reviewed in their entirety.Thirty-six articles met proposed inclusion criteria. These papers were divided into three categories— 23 in Surgical, nine in Anatomy, and four in Other. Twenty-two total ARAs were described: 15 in Surgical, five in Anatomy, and two in Other. Of the 36 included articles, 26 (72%) were published in the last five years and eight (22%) were published between 5-10 years ago. A PRISMA flowchart detailing this literature search is displayed in Figure 2.

**Figure:**
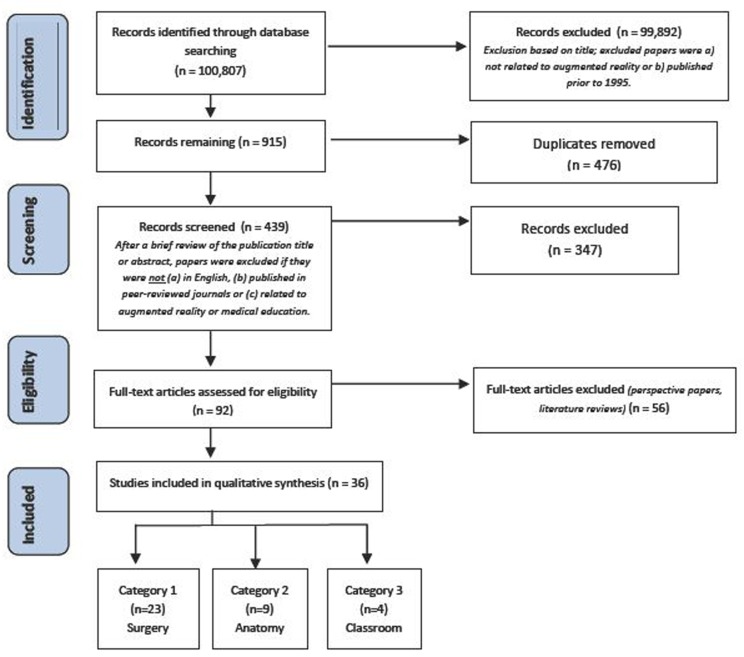
Figure 2.PRISMA flow diagram

Evaluation of study quality is delineated in Tables 1 and 2 (see Appendix A). Using the GRADE criteria, the majority of ARAs were graded low or very low quality. Only three of 22 ARAs (14%) received a quality grade above Low, and only one (4.5%) received a High rating.^23^^-^^25^ Points were primarily lost for study design, lack of data, and outcome inconsistency - seven (19%) of 36 articles were RCTs and twelve (33%) had sample sizes less than 50. Of the seven RCTs, six were given a Low or Moderate rating due to small sample sizes and inconsistent results. Only the ProMIS simulator, ImmersiveTouch, Microsoft Kinect ARMM test system, AR MagicBook, EyeSI, and mARble were evaluated by more than one study. Many ARAs have only been the subject of a single study (e.g. Google Glass, Microsoft Hololens, and the virtual patient (VP) DIANA) and thus remain largely untested.

Validity assessments were not performed for 11 of the included ARAs (50%) and no application achieved all five stages of validity.In the following sections, we describe in more detail the ARAs that have been evaluated by two or more studies. Tables 3 and 4 contain a full list of identified ARAs and associated study outcomes (see appendix B).

### Overview of well-studied ARAs

**Surgical applications:** ProMIS AR Laparoscopic Simulator (Haptica, Dublin, Ireland)

Of 36 studies, seven involved use of the ProMIS simulator. Composed of a torso-shaped mannequin connected to a computer, this device trains students in laparoscopic procedures and combines the benefits of haptic feedback with the ability to view simulation feedback videos. Three cameras within the mannequin identify inserted instruments from different angles. Substitution of the peritoneal cavity with plastic trays allows the simulator to be used for multiple tasks.

The ProMIS AR simulator was used to train users on sigmoid colectomies,^26^ suturing,^27^^-^^30^ and other basic laparoscopic tasks.^30^^-^^32^ Overall, the ProMIS trainer was an effective educational tool. It was described as highly realistic and improved task-effectiveness across all studies.^27^ Studies that measured the difference in skill between novice and experienced participants found a significant correlation between high performance metrics and experience, indicating that the ProMIS simulator is reliable for evaluation of laparoscopic skills.^26^^,^^28^^,^^30^^,^^32^ It is important to note that the majority of these studies were pilot studies with low numbers of participants (n=7-28) with the exception of one (n=115).^31^ Additionally, none of the studies were randomized, only one was controlled,^26^ and most depended on subjective means such as Likert-scale surveys to determine performance.

ImmersiveTouch System (ImmersiveTouch, Inc., University of Illinois, Chicago, IL, USA)

Another AR training simulator that provides haptic feedback is the ImmersiveTouch system. ImmersiveTouch involves the integration of a head-hand tracking system with a stereoscopic display and is typically used for neurosurgical training.

Two randomized controlled trials (RCTs) evaluated the ImmersiveTouch system -- one for thoracic screw placement^33^ and the other for ventriculostomies.^34^ Use of the ARA slightly lowered failure rate in screw placement and demonstrated a statistically significant improvement of correct catheter placement for ventriculostomies. However, these experiments had small sample sizes of 51 and 16 participants, respectively.

EyeSI AR Binocular Indirect Ophthalmoscopy (BIO) Simulator (VRmagic Holding AG, Mannheim, Germany)

The EyeSI AR simulator displays virtual retinae on a model head through a lens inspired by traditional BIO lenses. The user physically adjusts the lens to look in different directions while their movements are recorded on a separate monitor.

Two RCTs compared traditional BIO lenses to the EyeSI AR simulator. Rai et al. (n=28) randomized first-year ophthalmology residents to traditional and EyeSI training methods and evaluated their performance in three tasks.^35^ The AR group significantly outperformed the control group in both raw score and mean performance and was able to complete the procedure in less time. Leitritz et al. (n=37) randomized 4^th^ year medical students with no prior experience with BIO into control and AR groups using the EyeSI simulator.^36^ All students performed the procedure the day after training and were assessed through their drawings of the patient’s optic disk and arteries/veins. The AR group sketched more vessels correctly and achieved a higher Ophthalmoscopy Training Score.

***Anatomy*** ***applications:*** *AR Magic Book (various)*Several studies utilized a system called “MagicBook.”^37^^,^^38^ A number of specific ARAs fit into this category (see Table 4) but all consisted of a standard didactic textbook with cards for relevant anatomical figures. These cards could be recognized by a computer webcam or a smartphone and were able to display a virtual, interactive representation of the figure on the connected display.

Two large RCTs conducted by Ferrer-Torregrosa et al.^23^^,^^24^ concluded that this type of ARA improved attention, recall, learning, structure, imaging, and understanding in university students. The AR group scored significantly higher than the traditional learning control groups on final assessments. Most respondents believed that AR was effective for studying (76.9%), that it increased motivation and interest (75%), and that their grades would improve if professors utilized the technology (67.3%). Another RCT conducted by Kucuk et al.^25^ demonstrated similar results: medical students utilizing the “MagicBook” ARA scored significantly higher on an academic test with lower cognitive load compared to control and 100% of respondents reported that AR either greatly or partially facilitated learning.

*Microsoft Kinect (Microsoft Corp., Redmond, WA, USA)*The Microsoft Kinect was often used as part of an “AR Magic Mirror” (ARMM) approach. The Kinect contains a high-resolution camera for video reproduction and a low-resolution camera for depth perception, allowing the device to accurately track the user’s body movements. The system is often used for interactive video games but can be adapted to allow overlay of tracked virtual information onto a user’s body.

There were three papers exploring the ARMM application; all were surveys directed at medical students and clinicians.^39^^-^^41^ Responses from all three were positive. Varying majorities of respondents reported that ARMM increased learning motivation (58%), was beneficial in an educational setting (69.1%), stimulated active learning (82.4%), and improved 3-Dimensional understanding of anatomy (93.4%) while remaining easy to use.^39^^,^^40^ A large majority (80.5%) rated the system as excellent or good, and surveyed physicians unanimously recommended that ARMM be used to supplement existing anatomy curriculums.^41^

***Other*** ***applications:*** *Mobile AR Blended Learning Environment (mARble) [Peter L. Reichertz Institute for Medical Informatics at the Hannover Medical School, Hanover, Germany]*

The mARble is an application developed for the Apple mobile operating system that stores content separately from the program’s code; this allows for the addition of modules to adapt the application for different purposes without changing its source code. Three studies evaluated the mARble application; two were RCTs^42^^,^^43^ and one was a survey.^44^ All three had small sample sizes, with two recruiting ten or less participants.^42^^,^^44^ Students described the application as pragmatic and enjoyable to use, but the two RCTs yielded conflicting results. Albercht et al. concluded that mARble increased knowledge retention with lower cognitive fatigue when compared with traditional textbook material,^42^ but Noll et al. found no difference in knowledge gain between mARble and control groups immediately after training, although the AR group retained more knowledge in a follow-up assessment 14 days after training.^43^

### Analytical model

**Figure 3:**
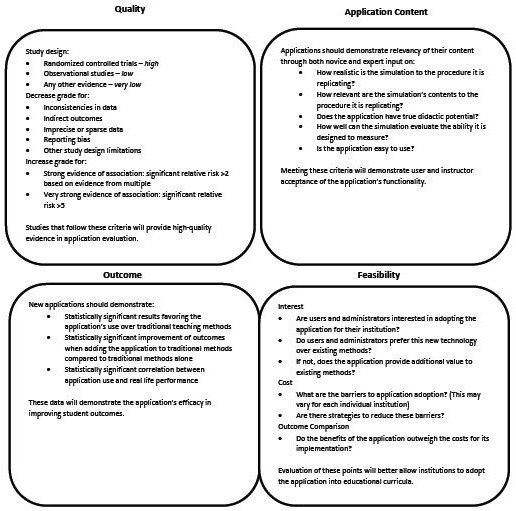
Augmented Reality Research Model for curricular integration

To address the low quality of most studies and the lack of standard ARA assessment, we developed an analytical model to evaluate the potential for an ARA to be integrated into a medical education curriculum. We divided this model into four primary components:*quality, application content, outcome*, and *feasibility* (Figure 3).

*Quality* references the caliber of study design and consistency of evidence.^15^ As recommended by GRADE criteria, future AR research should utilize more rigorous study designs and larger study sizes as well as conduct more studies on existing ARAs to provide further feedback and high-quality evidence supporting curricular integration. Importantly, subjective metrics such as “realism” proposed by GRADE criteria were not included in this model.

*Application content* refers to the quality and design of the application itself. Future ARAs should be designed to closely mimic or enhance the desired procedure/setting and should add value to the teaching experience. Furthermore, to be implemented in educational curricula, applications should provide feedback and be consumer-oriented. This may be assessed by both novices and experts in the area an ARA is designed to simulate. Positive user input on the points listed in Figure 2 demonstrate support by the ARA’s intended audience.

*Outcome* assesses the nature of study results: statistically significant values favoring ARA use over traditional teaching methods and positive user feedback on usability and didactic potential are both needed for strong outcomemetrics. While ARAs that successfully address ‘*Application Content*’ demonstrate qualitative support for curricular integration, ‘*Outcome*’ metrics provide additional quantitative support.

Finally, the *feasibility* module highlights the rarely-discussed factors of interest, cost, and ARA adoption outcomes. While this may be a topic better suited to entrepreneurs and application developers, future research should also understand the balance between an application’s value and its barriers to implementation. Many of the ARAs described in this article, such as the VP DIANA, were not designed for consumer or educator use and therefore have less potential for curricular integration. Developing consumer-oriented applications and maintaining industry awareness of the resources required for new technologies will inform program decisions and help ensure sustainability.^22^^,^^45^^,^^46^

Researchers interested in developing or testing new AR technology can address each of these four categories or provide a rationale for exclusion prior to implementing an ARA in a medical curriculum.

## Discussion

While AR technology has the potential to improve or replace some conventional medical training methods, this systematic review demonstrated inconsistency in both focus and quality of the published studies. Overall, most studies lacked validity assessments of their ARAs and were of low quality due to poor study design, small sample sizes, and inconsistent outcomes. Notably, half of all included articles were observational studies and 31% were surveys. While a randomized controlled trial is the highest-quality study type, the large percentage of subjective surveys significantly limited the impact of the research. Outside of providing evidence supporting face and content validity, surveys add little to the field in terms of promoting ARA implementation and should be used primarily as an adjunct to objective data in future studies.

Despite these shortcomings, many studies established positive responses toward AR and a desire by both trainees and experts to see the technology implemented in training programs. Furthermore, most articles identified in this systematic review were published within the last five years. Both of these findings underscore the increasing relevance and consumer interest in the application of AR simulation in medical education.

The state and quality of research varied widely between surgical, anatomical, and other ARAs. Surgical ARAs included a variety of laparoscopic simulators (ProMIS, ImmersiveTouch), AR glasses (Google Glass^©^, Microsoft Hololens^©^, etc.), and AR telementoring systems (ART, STAR). This diversity reflects the well-documented use of simulation as a surgical training tool.^9^^,^^47^^,^^48^ Surgical ARAs were more consistently tested for validity than applications in the other two categories, likely due to the surgical origins of modern validation techniques.^16^ Several articles aimed to demonstrate specific stages of validity. However, we contend that these tests of validity should be modified and adapted to all uses of AR in medical education. The development of AR hardware by leading technology corporations such as Google, Microsoft, Brother, and Epson also indicate the potential integration of consumer products into medical settings. While recent technological advances have made AR simulation more viable for surgical training, further developments will need to broaden in scope to focus on more than technical skill.^9^^,^^49^ A holistic approach to training effective surgeons will require the integration of knowledge and attitude education^50^^,^^51^ as well as development of standardized assessments of simulation training in the operating room.^52^^,^^53^

Anatomical ARAs generally used a “MagicBook” or ARMM approach. Augmented reality technology is easily applied to anatomy learning due to its heavy reliance on spatial and 3-dimensional conceptualization – a hallmark of digital simulation. Consequently, the use of digital technology to enhance anatomical learning has already been studied for over a decade.^54^ This extensive history is reflected by higher quality evidence: anatomical studies include several large RCTs, specifically for “MagicBook” experiences.^23^^-^^25^ Three studies found that the use of this technology significantly improved student assessment scores post-training, indicating reproducible potential and high quality evidence by GRADE criteria.^15^

Studies in the Other category did not offer compelling evidence for AR implementation. There was a lack of consistently positive outcomes and high-quality studies for both mARble^42^^-^^44^ and DIANA.^55^ Study sample sizes were also small. Outcomes of mARble were conflicting: Albrecht et al. concluded that mARble was superior to traditional textbook learning^42^ while Noll et al. demonstrated that mARble did not produce better knowledge retention than mobile phone applications.^43^ The VP DIANA produced worse assessment and empathy scores than traditional SP experiences.^55^ This may be a result of the unrealistic design of the system; adjustments to enhance the realism of the VP DIANA module and incorporation of more modern AR simulation technology (including AR glasses) may improve student outcomes.

The breadth of projects identified in this review highlights both the adaptability of AR technology and the lack of standardized assessment tools. Our analytical model (Figure 3) sought to address this discrepancy. Frameworks in medical education have been developed to analyze technology research^22^^,^^56^^,^^57^ but have not proposed a model to evaluate the readiness of educational AR tools for curricular implementation. The four categories introduced in our analytical model encompass the largest factors determining an ARA’s success in the medical classroom or operating room. Although the quality and validity metrics used in our systematic review only covered the criteria in three of four categories (*quality, application content*, and *outcome*), future studies should address all four categories (including *feasibility*) in order to thoroughly consider the key barriers to AR implementation. While we incorporated many aspects of Gallagher et al.’s validity framework into the *application content* module, we refrained from specifying which framework to use in validating ARAs as we believe the criteria should be adjusted and distinctively prioritized to reflect each application’s unique educational goals. We hope this model will encourage future studies to incorporate both higher quality study designs and formal validity assessments.

## Limitations

Our study has several limitations. An inevitable flaw in systematic reviews is the possibility of reporting bias due to search criteria (e.g., studies published in languages other than English, choice of keywords, scope, or databases).^58^ However, bias was minimized by using several independent reviewers and consulting with a science librarian. Given the rapid growth of AR technology in recent years, it is also probable that research involving certain cutting-edge applications have not yet been published or are under patent/copyright restrictions, precluding their inclusion in this review. Finally, many criteria put forth in this paper regarding study quality and training potential are inherently subjective and may not be broadly applicable to every program or student population.

### Conclusion

The use of AR technology in medical education is in its early stages presently lacks evidence-based support for its widespread implementation. Future research should adopt long-term and large-scale RCT or cohort study designs in keeping with the proposed model to evaluate ARA efficacy. Rigorous and standardized validation of commercially viable applications will allow the technology to be more readily integrated into medical educational curricula.

## Appendix A

**Table 1:** Quality assessment of surgery augmented reality applications

Application (# of studies)	Design	Purpose	Stages of Validity Tested ^€^					Quality^§^
			1	2	3	4	5	
ProMIS (7)	Observational study (6) Survey (1)	Basic laparoscopic skills	x		x			Moderate
		Suturing	x		x	x		
		Laparoscopic colectomy						
ImmersiveTouch (2)	Observational studies	Ventriculostomy	x					Low
		Thoracic pedicle screw placement						
ARToolKit (1)	Observational study	Echocardiography						Very low
Vuzix 920AR (1)	Observational study	Tumor resection planning						Very low
STAR (1)	Observational study	Surgical telementoring						Very low
Brother AiRScouter WD-200B (1)	RCT	Central line insertion						Low
EyeSI (2)	RCTs	Binocular indirect ophthalmoscopy	x		x	x		Moderate
HoST UVA (1)	RCT	Urethrovesical anastomosis	x			x		Low
Google Glass (1)	Survey	Inflatable penile prosthesis placement						Very low
Prototype simulator (1)	Survey	Ultrasound-guided needle placement						Very low
Epson Moverio BT-200 (1)	Survey	Central line insertion	x					Very low
MicronTracker 2 (1)	Survey	Spinal needle insertion	x					Very low
ART (1)	RCT	Surgical telementoring	x			x		Low
Microsoft Hololens (1)	Observational study	Surgical telementoring	x			x		Very low
FLS (1)	Observational study	Peg transfer task						Very low

€ For stages of validity, see Figure 1

§ Quality rank based on GRADE guidelines^15^

**Table 2:** Quality assessment of anatomy and other augmented reality applications

Application (# of studies)	Design	Stages of Validity Tested ^€^					Quality^§^
		1	2	3	4	5	
**Anatomy**							
Unspecified application (1)	Survey						Very low
AR Magic Books (3)	RCTs				x		High
AR Magic Mirror (3)	Surveys	x	x				Low
Unity v5 (1)	Observational study				x		Low
BARETA (1)	Survey						Very low
**Other**							
mARble (3)	RCTs (2) Survey (1)						Low
DIANA virtual patient (1)	RCT						Low

€ For stages of validity, see Figure 1

§ Quality rank based on GRADE guidelines^15^

## Appendix B

**Table 3:** Augmented reality applications in surgery

Augmented reality application	Sample size	Purpose	Outcome
ProMIS Augmented Reality Laparoscopic Simulator (Haptica, Dublin, Ireland)	55	Laparoscopic skills	Realism considered good to excellent by all participants, mixed evaluations of didactic value^27^
	18	Suturing	Significant improvement in knot scores following training with the simulator^29^
	15	Laparoscopic skills	Improvement in task completion with greater efficiency^32^
	46	Laparoscopic skills	Significant correlation between experience and performance^30^
	24	Suturing	Experienced participants had higher performance scores than novice participants^28^
	35	Laparoscopic colectomy	Simulator model rated as easier than cadaver model^26^
	115	Laparoscopic skills	Experience levels correlated strongly with simulation scores^31^
ImmersiveTouch System (ImmersiveTouch, Inc., University of Illinois, Chicago, IL, USA)	16	Ventriculostomy	AR group more likely to succeed on first attempt. Residents praised the simulator for its realism^34^
	51	Thoracic pedicle screw placement	Non-significant reduction of failure rate in screw placement^33^
ARToolKit (ARToolWorks Inc., Seattle, WA, USA)	10	Echocardiography	Trainees were able to successfully perform an ECG test^59^
Vuzix 920AR goggles (Vuzix Corp., Rochester, NY, USA)	21	Tumor resection planning	Improved non-clinicians’ performance and significantly improved time to task completion for clinicians^60^
System for Telementoring with Augmented Reality (STAR) [Purdue University, West Lafarette, IN, USA]	20	Surgical telementoring	Less placement errors and fewer focus shifts, but took more time for each task^61^
Brother AiRScouter WD-200B AR glasses (Brother International Corp., Bridgewater, NJ, USA)	32	Central line insertion	No difference in median total procedure time between AR and control groups^62^
EyeSi augmented reality binocular indirect ophthalmoscopy simulator (VYmagic Holding AG, Mannheim, Germany)	28	Binocular indirect ophthalmoscopy (BIO)	AR group demonstrated superior total scores and performance^35^
	37	BIO	More correct sketched vessels and higher Ophthalmoscopy Training Score for AR group^36^
Hand-on Surgical Training (HoST) urethrovesical anastomosis (UVA) AR module (Roswell Park Cancer Institute and the State University of New York at Buffalo Virtual Reality Laboratory, New York, NY, USA)	52	UVA	HoST group outperformed control group on multiple measures while having lower temporal demand and mental fatigue^63^
Google Glass (Google Inc., Mountain View, CA, USA)	30	Inflatable penile prosthesis placement	81% of participants recommended implementation of application into training program; 93% felt Google Glass has a place in the operating room^64^
Unspecified prototype AR simulator	60	Ultrasound-guided needle placement	Majority positive responses for usability and training feasibility^65^
Epson Moverio BT-200 Smart Glasses (Epson America, Inc., Long Beach, CA, USA)	40	Central line insertion	Participants reported that simulation was realistic, easy to use and useful for training; 59.3% responded that AR was better than other training methods^66^
MicronTracker2 (Claron Technologies, Toronto, ON, Canada)	10	Spinal needle insertion	Overall positive responses to the system by trainees^67^
Augmented reality telementoring (ART) platform (University of Nevada School of Medicine, Las Vegas, NV, USA)	18	Surgical telementoring	After training, ART group was faster and had fewer failed attempts^68^
Microsoft Hololens (Microsoft Corp., Redmond, WA, USA)	24	Surgical telementoring	Mixed feedback on Hololens versus full telemedicine setup, no statistical difference in performance^69^
Fundamentals of Laparoscopic Surgery (FLS) module (Society of American Gastrointestinal and Endoscopic Surgeons, Los Angeles, CA, USA)	20	Standard peg transfer	Participants preferred using the timed overlay over no feedback; no difference in time to task completion or muscle fatigue^70^

**Table 4:** Anatomy and other augmented reality applications

Augmented reality application	Sample size	Outcome
**Anatomy**		
Unspecified ARA	28	Positive responses for understandability and ease of use; most (70%) felt it was useful in anatomy education^71^
AR Magic Books (Various)	211	AR group scored significantly better on final assessment; most participants responded positively to AR^24^
	70	AR group scored significantly higher on academic test with lower cognitive load; all participants reported that AR facilitated learning^25^
	171	AR group had significantly higher scores than the video and notes groups; 76.9% of participants considered AR effective for studying^23^
AR Magic Mirror (ARMM) system using Microsoft Kinect (Microsoft Corp., Redmond, WA, USA)	748	Majority responded that ARMM stimulated active learning and improved structural understanding^39^
	79	Majority positive responses (80%)^41^
	68	Majority (82%) reported that ARMM facilitated knowledge retention and was easy to use^40^
Unity v5 (Unity Technologies ApS, San Francisco, CA, USA)	59	No significant difference in test scores between AR, VR and 3D modeling groups^72^
Bangor Augmented Reality Education Tool for Anatomy (BARETA) [Bangor University, Bangor, Gwynedd, UK]	34	Majority reported that BARETA helped them learn anatomical structures and was easier to use than a mouse-and-keyboard interface^73^
**Other**		
Mobile AR blended learning environment (mARble) [Peter L. Reichertz Institute for Medical Informatics at the Hannover Medical School, Hanover, Germany]	44	AR group scored slightly higher in post-training exam but had lower knowledge retention at 14 days^43^
	10	AR group scored slightly higher in post-training exam with lower cognitive load and significantly higher hedonistic scores^42^
	6	Pragmatic quality of mARble was rated averagely, while hedonic aspects were rated above average^44^
Digital Animated Avator (DIANA) virtual patient (Medical College of Georgia, Augusta, GA, USA)	84	AR group scored significantly lower in empathy and overall rating^55^
